# Influence of H19 polymorphisms on breast cancer: risk assessment and prognostic implications via LincRNA H19/miR-675 and downstream pathways

**DOI:** 10.3389/fonc.2024.1436874

**Published:** 2024-08-29

**Authors:** Ying Qi, Pengfei Zhao

**Affiliations:** ^1^ Department of Radiology, Shengjing Hospital of China Medical University, Shenyang, China; ^2^ Department of Pharmacology, School of Pharmaceutical Sciences, China Medical University, Shenyang, China

**Keywords:** H19, Mir675, breast cancer, genetic polymorphism, susceptibility, prognosis

## Abstract

**Introduction:**

Breast cancer, as the most prevalent malignancy among women globally, continues to exhibit rising incidence rates, particularly in China. The disease predominantly affects women aged 40 to 60 and is influenced by both genetic and environmental factors. This study focuses on the role of H19 gene polymorphisms, investigating their impact on breast cancer susceptibility, clinical outcomes, and response to treatment.

**Methods:**

We engaged 581 breast cancer patients and 558 healthy controls, using TaqMan assays and DNA sequencing to determine genotypes at specific loci (rs11042167, rs2071095, rs2251375). We employed *in situ* hybridization and immunohistochemistry to measure the expression levels of LincRNA H19, miR-675, MRP3, HOXA1, and MMP16 in formalin-fixed, paraffin-embedded samples. Statistical analyses included chi-squared tests, logistic regression, and Kaplan-Meier survival curves to evaluate associations between genetic variations, gene expression, and clinical outcomes.

**Results:**

Genotypes AG at rs11042167, GT at rs2071095, and AC at rs2251375 were significantly associated with increased risk of breast cancer. Notably, the AA genotype at rs11042167 and TT genotype at rs2071095 were linked to favorable prognosis. High expression levels of LincRNA H19, miR-675, MRP3, HOXA1, and MMP16 in cancer tissues correlated with advanced disease stages and poorer survival rates. Spearman correlation analysis revealed significant positive correlations between the expression of LincRNA H19 and miR-675 and specific genotypes, highlighting their potential regulatory roles in tumor progression.

**Discussion:**

The study underscores the critical roles of LincRNA H19 and miR-675 as prognostic biomarkers in breast cancer, with their overexpression associated with disease progression and adverse outcomes. The H19/LincRNA H19/miR-675/MRP3-HOXA1-MMP16 axis offers promising targets for new therapeutic strategies, reflecting the complex interplay between genetic markers and breast cancer pathology.

**Conclusion:**

The findings confirm that certain H19 SNPs are associated with heightened breast cancer risk and that the expression profiles of related genetic markers can significantly influence prognosis and treatment response. These biomarkers hold potential as targets for personalized therapy and early detection strategies in breast cancer, underscoring the importance of genetic research in understanding and managing this disease.

## Introduction

Breast cancer is a common malignant tumor in women, with 2.26 million women diagnosed and 685,000 deaths in 2020 (1). Statistics from multiple cities in China also show that breast cancer is the most common malignant tumor in Chinese women, and its incidence is increasing yearly (2). Epidemiological studies have shown that breast cancer mostly occurs in women aged between 40 and 60, especially before and after menopause (3). The occurrence and development of breast cancer is thought to be the result of the combined effects of environmental and genetic factors (4). Improving breast cancer screening in high-risk groups, reducing the incidence of breast cancer and improving the cure rate are challenges that need to be addressed (5). The risk of breast cancer has been confirmed to be closely associated with mutations and expression changes in genes such as BRCA1, BRCA2, P53, epidermal growth factor receptor (EGFR), and Ki-67 (6–8).

Human genes have two copies (alleles), one inherited from the father and the other from the mother. For most genes, the two copies are equally expressed. However, at some loci, expression is determined by parental origin, and only the copy from one parent is expressed. This parent-of-origin-dependent differential expression phenomenon is called genomic imprinting. Genes that are differentially expressed under imprinting regulation are called imprinted genes. The human H19 gene is located in the imprinted gene cluster of chromosome 11p15.5. The paternal allele is highly methylated and not expressed, whereas the maternal allele is unmethylated and expressed (9). The gene contains five exons and four introns, and the transcription products of the H19 gene are a 2.3 kb RNA molecule and a small molecule, miR-675, both of which lack open reading frames and do not encode proteins. Hence, they are called noncoding RNAs (10, 11). Noncoding RNAs generally play regulatory roles in genes at the transcriptional, posttranscriptional, and epigenetic levels (12). Although H19 RNA molecules can be detected in both the cytoplasm and the nucleus, they exist mainly in the cytoplasm, where they function as regulatory RNAs or riboregulators (13). New studies have found that H19, an imprinted gene associated with the occurrence and development of various tumors, may also be one of the risk factors for breast cancer (14). Genetic studies have shown that the promoter and intron regions of the H19 gene are involved in gene expression regulation. Key site mutations in these regions can directly affect the expression of targeted genes, thereby participating in the occurrence, development, and drug treatment efficacy of diseases (15).

This suggests that finding and verifying the H19 polymorphisms that are closely related to breast cancer is of great significance. Single nucleotide polymorphisms (SNPs) are the most common type of genetic variation, accounting for more than 90% of known polymorphisms; approximately one SNP is present in every 1,000 base pairs (16). The study of SNPs helps to explain differences in individual susceptibility to diseases (17), differences in drug tolerance (18), and differences in reactions to environmental factors (19). The study of polymorphisms in the H19 gene may help to uncover the relationship between breast cancer and H19 (9). Additionally, LincRNA H19 has been confirmed to endogenously generate miR-675 (20). Studies indicate that the aberrant expression of LncRNA H19 and its derivative miR-675 is closely associated with the occurrence, development, and clinical prognosis of various tumors (21–24). In tumor tissues, variations in LincRNA H19 expression correlate positively with changes in miR-675 expression. This interaction orchestrates the regulation of downstream target genes, leading to the emergence of resistance, invasion, and metastasis in cancer cells (25–27).

**Figure 1:**
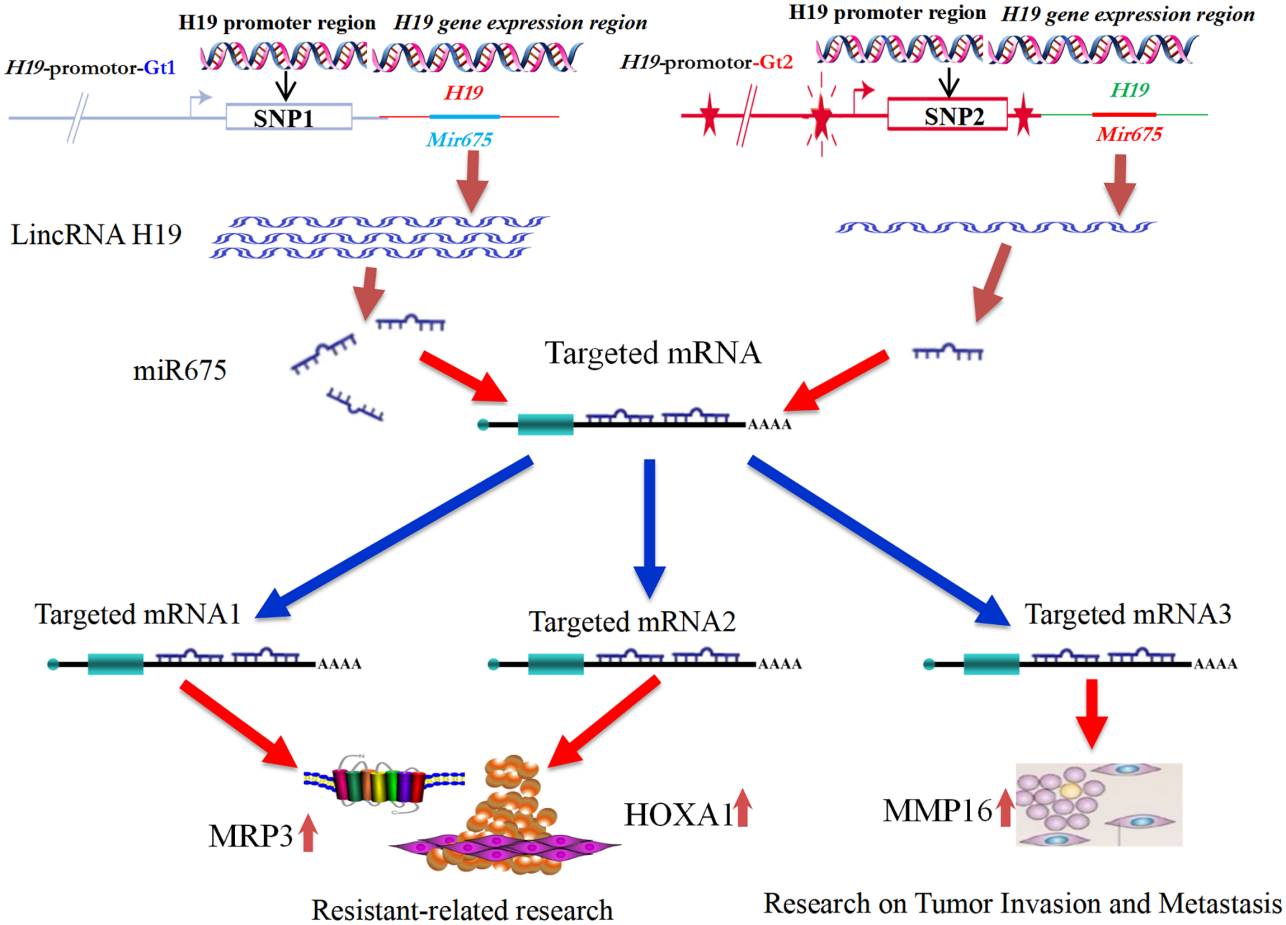
Diagram of the LincRNA H19/miR-675 and MRP3-HOXA1-MMP16 Pathways Influencing the Pathogenesis and Progression of Breast Cancer.

This study explored the role of H19 polymorphisms in breast cancer, assessing their impact on risk, clinical outcomes, treatment responses, and prognosis via SNP analysis. We posited that increased levels of LincRNA H19 and miR-675 in breast cancer biopsies could serve as effective prognostic biomarkers. Using *in situ* hybridization and immunohistochemistry, we measured the expression of LincRNA H19, miR-675, MRP3, HOXA1, and MMP16 in formalin-fixed, paraffin-embedded samples. Our findings indicate that LincRNA H19 and miR-675 are reliable indicators of patient outcomes. The expression profiles suggest that the H19/LincRNA H19/miR-675/MRP3-HOXA1-MMP16 axis contributes to the initiation and progression of breast cancer, highlighting potential targets for therapeutic strategies (refer to **Figure 1**).

## Materials and methods

### Study subjects and specimen collection

This study involved 581 breast cancer patients and 558 healthy controls, all Chinese women. The Ethics Committee of Shengjing Hospital at China Medical University approved the study protocol (IRB2018PS132J). Written informed consent was waived as data were extracted from clinical records. Patients were recruited from the Oncology and Breast Surgery Departments at The First Hospital of China Medical University and the Breast Surgery Department at Shengjing Hospital, between September 2003 and July 2013. All participants were newly diagnosed with tumors verified by histopathology, had not undergone any anticancer treatments prior to joining the study, and had comprehensive medical records ensuring the reliability of clinical data.

The healthy control group comprised individuals from routine health examinations at the Health Examination Center of The First Hospital of China Medical University between September 2003 and July 2013. This group included unrelated, healthy female volunteers without tumors or genetic diseases. Criteria for inclusion were: absence of any tumors (benign or malignant), age comparable to the breast cancer patient group, and no significant medical conditions as determined by examination results.

After obtaining informed consent, 2 mL of fasting peripheral venous blood was drawn from both breast cancer patients and healthy volunteers into vacuum blood collection tubes with EDTA-2Na^+^ anticoagulants. The samples were then stored at −80°C for subsequent analysis. Breast cancer tissue microarray chips were prepared from previously diagnosed patients, using adjacent non-cancerous tissue as controls.

### Experimental methods

#### SNP site selection for the TaqMan^®^ assay

We employed dual-labeled TaqMan probes, one with FAM and the other with HEX, to distinguish between genotypes at specific SNP loci using a multiplex PCR assay. Selection of SNPs was guided by criteria from the NCBI dbSNP database, specifically choosing SNPs with a heterozygosity rate above 8% in the Chinese population and those validated for TaqMan assays, as per the Life Technologies website. Consequently, we targeted rs11042167, rs2071095, and rs2251375 SNPs in the H19 gene promoter for this study.

#### TaqMan fluorescent probe SNP genotyping method

We extracted genomic DNA from peripheral blood leukocytes using the KI method and performed SNP genotyping via the TaqMan assay. Specific TaqMan probes used for allelic discrimination included: rs11042167 labeled with HEX for the A allele and FAM for the G allele; rs2071095 labeled with HEX for the G allele and FAM for the T allele; and rs2251375 labeled with HEX for the C allele and FAM for the G allele. Detailed probe sequences are provided in the **Supplementary Material**. PCR was conducted on an Applied Biosystems MX3000p Real-time PCR system, starting with a 30-second denaturation at 95°C, followed by 45 cycles of denaturation at 95°C for 5 seconds and annealing at 60°C for 20 seconds, with fluorescence measurements taken post each cycle. Components of the PCR mix are detailed in **Table 1**. The PCR products were sequenced by Sangon Biotech, Shanghai.

**Table 1:** Taqman real-time PCR reaction system.

reagent	1×Enzyme digestion reaction system/µL
Taqman Assay	1.25
2×Premix Ex Taq MIX	2.5
DNA	1.0
deionized water	0.5
total volume	5.0

#### *In Situ* hybridization experiments and interpretation standards

The *in situ* hybridization kit, purchased from Exiqon, Denmark, involves a standard procedure starting with routine deparaffinization of the tissue microarrays. The samples are then digested at 37°C with 3% freshly diluted citric acid pepsin for 15 minutes, followed by a gradient ethanol dehydration. Probes for LincRNA H19 or miR675, labeled with digoxigenin, are pre-hybridized at 55°C for 2 hours. After removing the excess liquid without washing, the probes are hybridized overnight. Post-hybridization washing is done with SSC, followed by blocking solution at 37°C for 30 minutes without subsequent washing. Biotinylated mouse anti-digoxigenin antibodies are incubated at room temperature for 120 minutes. This is followed by the addition of SABC for 30 minutes at room temperature and three PBST washes for 5 minutes each. Biotinylated peroxidase is added and incubated at room temperature for 30 minutes, followed by four PBS washes for 5 minutes each. The samples are then stained with DAB, counterstained with hematoxylin, thoroughly washed with water, dehydrated with alcohol, cleared in xylene, and finally coverslipped. Omission of the probe serves as a negative control. The results are determined by the brown-yellow granular positive signals of H19 and miR675 located in the cytoplasm and nuclei of breast ductal epithelium or cancer cells. The signal intensity for H19 and miR675 is scored from 0 to 4, with 0–1 indicating no signal, >1–2 a weak signal, >2–3 a moderate signal, and >3–4 a strong signal. Under high magnification (×400), 100 cells per field are counted in 10 random fields per slide to calculate the percentage of positive cells. The positivity rates are classified as 0, <25%, 25%–50%, 51%–75%, >75%, corresponding to scores of 0, 1, 2, 3, and 4, respectively. A product score of 0–1 indicates negative expression (–), >1–6 indicates positive expression (+), and >6 indicates strong positive expression (++). The tissue microarray results are independently reviewed and scored by three pathologists.

In this study, we employed *in situ* hybridization (ISH) to analyze the expression of miR-675 in breast cancer tissues and adjacent non-tumor tissues. We used probes targeting the overall sequence of miR-675, without specifically distinguishing between its mature isoforms, miR-675-5p and miR-675-3p. This decision was based on our primary objective to assess the role of miR-675 as a whole in the pathological processes, rather than to differentiate the specific contributions of its isoforms. The ISH procedure included the use of DIG-labeled LNA probes for hybridization. The probe design was specific to the mature sequence of miR-675 but did not differentiate between the 5p and 3p isoforms. Following hybridization, anti-DIG antibodies were used for signal detection. The final results were assessed under a microscope based on the intensity and distribution of the staining.

#### Immunohistochemistry testing and interpretation standards

After formaldehyde fixation and paraffin embedding, tissue sections are cut thickly. The procedure includes routine deparaffinization and rehydration, followed by peroxidase blocking with hydrogen peroxide in deionized water. Antigen retrieval is performed using EDTA at high temperatures, then cooled naturally and rinsed with PBS. Each section is treated with 100μl of primary antibodies: mouse anti-human MRP3 monoclonal, rabbit anti-human HOXA1 polyclonal, and mouse anti-human MMP-16 monoclonal, all diluted 1:100. After applying secondary antibodies, the sections are developed with DAB, counterstained with hematoxylin, dehydrated, cleared in xylene, and mounted with neutral resin. PBS replaces the primary antibody in the negative control. Positive results are semi-quantitatively determined using brown-colored HMGA2 particles as a positive marker. Staining intensity is graded on a scale from 0 (no color) to 3 (dark brown). Under high magnification (×400), 100 cells per field are counted across 10 random fields per slide to calculate the percentage of positive cells. Cell positivity rates are scored from 0 (no positive cells) to 4 (>75% positive cells). Total scores range from 0–1 points indicating negative expression (–), >1–6 points indicating positive expression (+), and >6 points indicating strong positive expression (++). The tissue microarray results are independently reviewed and scored by three pathologists.

### Establishment of a database and statistical analysis

We developed databases using Excel to manage genotype frequencies for rs11042167, rs2071095, and rs2251375, and expression data for LincRNA H19, miR-675, MRP3, HOXA1, and MMP16 for both case and control groups. Additional data on demographic and clinical characteristics including age, menopausal status, family history, pathology type, clinical stage, ER/PR/HER2 status, P53/BRCA1/BRCA2 status, lymph node metastasis, survival status, and treatment modalities were also cataloged.

Data were formatted for compatibility with various analytical software. We employed chi-squared tests to assess differences in sex and age between groups and to compare genotype frequencies and histological data. Hardy-Weinberg equilibrium for control group genotype frequencies was tested using Arlequin software, considering distributions in equilibrium at P > 0.05. Relative risks were quantified using a nonconditioned logistic regression model to generate odds ratios (ORs) and 95% confidence intervals (CIs). Kaplan-Meier survival analysis was used to estimate survival outcomes of breast cancer patients. All statistical analyses were performed using SPSS 29.0 and Python 3.10.

## Results

### Basic characteristics of the study subjects

This study involved 581 female breast cancer patients and 558 healthy female controls, matched by age and sex. The average age of the breast cancer group was 50 years (range: 22–85; median: 50), while the controls averaged 49 years (range: 23–70; median: 49). An independent sample t-test confirmed no significant age differences between the groups (P > 0.05). Detailed demographic data, risk factors, and clinical variables for both groups are presented in **Table 2**.

**Table 2:** Clinical characteristics of breast cancer patients (n=581) and healthy female subjects (n=558).

Characteristic	Cases		Controls	
	n	%	n	%
**Total number**	581	100	558	100
**Mean age (range, yrs)**	50 (22~85)		49 (23–70)	
Age, yrs				
<50	308	53.0	297	53.2
≥50	273	42.0	261	46.8
Menopausal status				
Premenopausal	315	54.2	305	54.7
Postmenopausal	266	45.8	253	45.3
Tumor size				
≤ 2.0 cm	251	43.2		
2.1-5.0 cm	236	40.6		
>5.0 cm	94	16.2		
Histology				
IDC	499	85.9		
ILC	22	3.8		
Others^b^	60	10.3		
Clinical stages				
I or II	472	81.2		
III or IV	109	18.8		
Lymph node metastasis				
Node-negative	326	56.1		
Node-positive	255	43.9		
ER status				
Negative	247	42.5		
Positive	326	56.1		
Undetermined	8	1.4		
PR status				
Negative	233	40.2		
Positive	339	58.3		
Undetermined	9	1.5		
HER2 status				
Negative	301	51.8		
Positive	265	45.6		
Undetermined	15	2.6		
TNBC status				
Yes	88	15.1		
No	485	83.5		
Undetermined	8	1.4		
p53 status				
Negative	254	43.7		
Positive	306	52.7		
Undetermined	21	3.6		
BRCA1 status				
Negative	111	19.1		
Positive	439	75.6		
Undetermined	31	5.3		
BRCA2 status				
Negative	219	37.7		
Positive	317	54.6		
Undetermined	45	7.7		
Therapeutic regimens				
Anthracycline-based chemotherapy^c^	355	61.1		
Paclitaxel-based chemotherapy^d^	47	8.1		
Anthracycline+paclitaxel-based chemotherapy^e^	47	8.1		
Other chemotherapies or treatments^f^	132	22.7		

IDC, Ivasive ductal carcinoma; ILC, Invasive lobular carcinoma; ER, Estrogen receptor; PR, Progesterone receptor; HER2, Human epidermal growth factor receptor 2; p53,Tumor suppressor protein 53; BRCA1, Breast carcinoma type 1 susceptibility protein; BRCA2, Breast carcinoma type 2 susceptibility protein.

^b^Others contains: invasive cribriform carcinoma, medullary carcinoma, and invasive papillary carcinoma.

^c^Chemotherapy of anthracycline-based contains: CE (Cyclophosphamide, Epirubicin); CA (Cyclophosphamide, Adriamycin); CEF (Cyclophosphamide, Epirubicin and 5-Fluorouracil); CAF(Cyclophosphamide, Adriamycin and 5-Fluorouracil).

^d^Chemotherapy of paclitaxel-based contains: Docetaxel or Paclitaxel and/or Capecitabine (T or TC regimens).

^e^Chemotherapy of anthracycline plus paclitaxel-based contains: CE or CA plus T regimens; CEF or CAF plus T regimens.

^f^Other chemotherapies or treatments included: CMF (Cyclophosphamide, Methotrexate and 5-Fluorouracil); C or CP, P or GP (Cyclophosphamide or Cyclophosphamide and Platinum, Platinum or Gemcitabine and cisplatin); NP, NX, or X (Navelbine and Platinum; Navelbine and Xeloda, Xeloda alone); 5-FU (5-Fluorouracil); Surgery only; radiation therapy and biological treatments or Chinese traditional treatment.

### TaqMan genotyping

**Figure 2:**
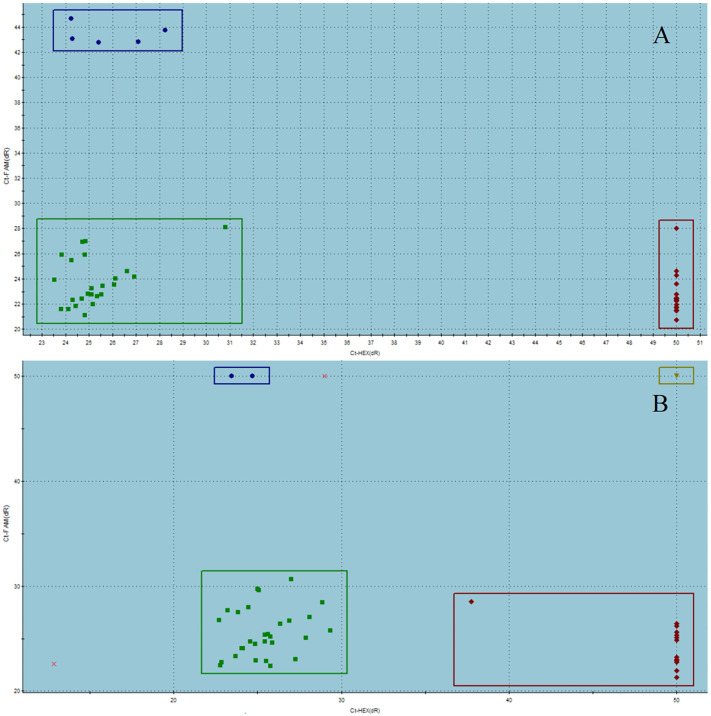
Fluorescence signal characteristics of genotypes in the control group **(A)** and the case group **(B)**.

Genotypes were identified using TaqMan real-time PCR based on changes in fluorescence intensity. The homozygous genotypes AA/GG/CC (rs11042167/rs2071095/rs2251375) displayed a significant increase in fluorescence in the FAM channel with no corresponding increase in the VIC (HEX) channel. Conversely, the homozygous genotypes GG/TT/GG (rs11042167/rs2071095/rs2251375) exhibited a significant increase in the VIC (HEX) channel without an increase in the FAM channel. The heterozygous genotypes AG/GT/CG (rs11042167/rs2071095/rs2251375) showed increased fluorescence in both FAM and VIC (HEX) channels. These findings are illustrated in **Figure 2**.

### Sequencing results for the amplification products of the H19 rs11042167, rs2071095, and rs2251375 SNPs

**Figure 3:**
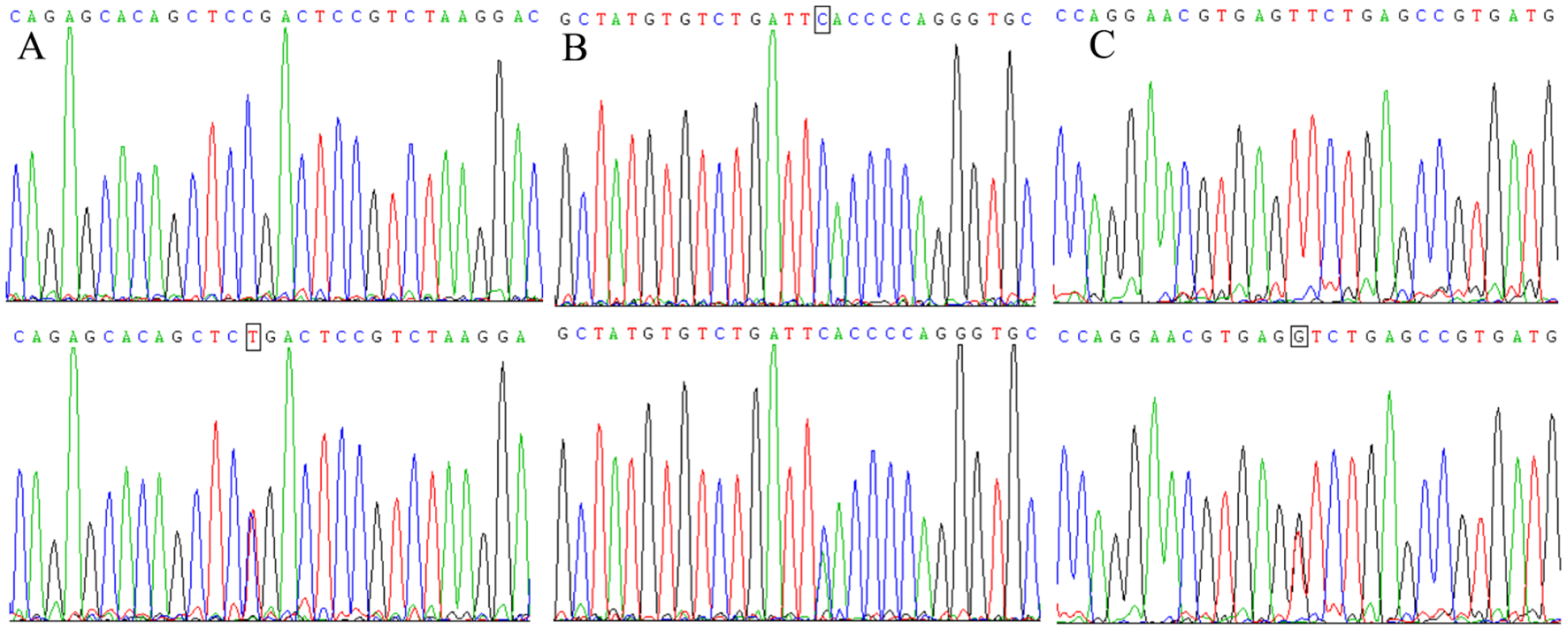
Genotyping sequencing results for rs11042167 **(A)**, rs2071095 **(B)**, and rs2251375 **(C)** SNPs.

**Figure 3** presents the sequencing results for specific genotypes at three SNPs: GG/GA at rs11042167, GG/GT at rs2071095, and AA/AC at rs2251375. The sequences, shown from the complementary strand in a 5′ to 3′ direction, illustrate that the homozygous genotypes produce a single peak at the nucleotide position, while heterozygous genotypes result in a double peak.

### Correlation between the H19 rs11042167, rs2071095, and rs2251375 SNPs and susceptibility to breast cancer

**Table 3** illustrates the genotype distributions for SNPs rs11042167, rs2071095, and rs2251375 in both breast cancer cases and controls. For rs11042167, the case group comprised 40 (6.9%) AA, 310 (53.3%) AG, and 231 (39.8%) GG genotypes, while the control group showed 54 (9.7%) AA, 209 (37.4%) AG, and 295 (52.9%) GG genotypes. Chi-squared testing confirmed Hardy-Weinberg equilibrium in the control group for rs11042167 (P > 0.05).For the rs2071095 SNP, the case group exhibited a genotype distribution of 231 cases (39.8%) with homozygous GG, 310 cases (53.3%) with heterozygous GT, and 40 cases (6.9%) with homozygous TT. In comparison, the control group showed 286 cases (54.6%) with homozygous GG, 227 cases (43.3%) with heterozygous GT, and 63 cases (12.0%) with homozygous TT. Chi-squared analysis confirmed that the genotype distribution in the control group was in Hardy-Weinberg equilibrium (P > 0.05). For SNP rs2251375, the case group presented 92 cases (15.8%) with homozygous AA, 366 cases (63.0%) with heterozygous AC, and 123 cases (21.2%) with homozygous CC. In the control group, there were 114 cases (20.4%) with homozygous AA, 271 cases (48.6%) with heterozygous AC, and 173 cases (31.0%) with homozygous CC. Chi-squared testing confirmed that the genotype distribution for rs2251375 in the control group was in Hardy-Weinberg equilibrium (P > 0.05).

**Table 3:** Frequency distribution of *H19* genotypes and their associations with the risk of developing breast cancer.

Genotypes	Controls ^†^ n (%)	Cases n (%)	*P* ^‡^	Adjusted OR (95% CI) ^§^
**All patients**	558 (100)	581 (100)		
*r*s11042167 (A→G)				
AA	54 (9.7)	40 (6.9)		1 (Reference)
AG	209 (37.4)	310 (53.3)	**0.002**	2.002 (1.283-3.124)
GG	295 (52.9)	231 (39.8)	0.806	1.057 (0.678-1.647)
AG/GG	469 (90.3)	541 (93.1)	0.087	1.449 (0.946-2.220)
A	320 (28.7)	390 (33.6)		1 (Reference)
G	728 (65.3)	772 (66.4)	**0.008**	**0.785 (0.657-0.939)**
rs2071095( G→T)				
GG	286 (54.6)	231 (39.8)		1 (Reference)
GT	227 (43.3)	310 (53.3)	**<0.001**	**1.584 (1.239-2.025)**
TT	63 (12.0)	40 (6.9)	0.166	0.737 (0.477-1.136)
GT/TT	290 (55.3)	350 (60.2)	**0.005**	**1.400 (1.107-1.771)**
G	799 (76.2)	772 (66.4)		1 (Reference)
T	353 (33.7)	390 (33.6)	0.325	1.092 (0.916-1.301)
rs2251375(A→C)				
AA	114 (20.4)	92 (15.8)		1 (Reference)
AC	271 (48.6)	366 (63.0)	**0.001**	**1.674 (1.220-2.296)**
CC	173 (31.0)	123 (21.2)	0.489	0.881 (0.615-1.262)
AC/CC	444 (79.6)	489 (84.2)	**0.044**	**1.365 (1.008-1.848)**
A	499 (44.7)	550 (47.3)		1 (Reference)
C	619 (55.3)	612 (52.7)	0.209	0.900 (0.763-1.061)

H19, H19 gene; H19,, Odds ratio; CI, Confidence interval.

The significance levels are P< 0.05 for all the bold values.

^†^The observed genotype frequency among individuals in the control group was in agreement with Hardy-Weinberg equilibrium.

^‡^P values were calculated from 2-sided chi-square tests for either genotype distribution or allele frequency.

^§^Adjusted OR and 95% CI values were calculated by unconditional logistic regression adjusted for age, menopausal state.

**Table 3** also presents the age-adjusted odds ratios (ORs) for genotypes at SNPs rs11042167, rs2071095, and rs2251375. For rs11042167, the OR for the AG genotype is 2.002 (95% CI: 1.283–3.124, P = 0.002), indicating a higher disease risk compared to the AA genotype. The GG genotype does not show an increased risk (OR = 1.057, 95% CI: 0.678–1.647, P = 0.806). At rs2071095, individuals with the GT genotype have an increased risk (OR = 1.584, 95% CI: 1.239–2.025, P < 0.001) compared to those with the GG genotype, while the TT genotype shows no increase (OR = 0.737, 95% CI: 0.477–1.136, P = 0.166). For rs2251375, the AC genotype is associated with a higher risk (OR = 1.674, 95% CI: 1.220–2.296, P = 0.001) relative to the AA genotype. Conversely, the CC genotype does not confer an increased risk (OR = 0.881, 95% CI: 0.615–1.262, P = 0.489).

### Correlation analysis between rs11042167, rs2071095, and rs2251375 SNPs and clinical pathological parameters of breast cancer patients

Pearson’s χ^2^ test and corrective measures (age, menopausal status, and family history) were utilized, alongside unconditional logistic regression analysis, to explore the relationship between the genotypes of the H19 SNPs and clinical pathological parameters of breast cancer patients. The data are presented in **Table 4**. Statistical analysis did not demonstrate any noteworthy frequency distribution differences between different genotypes of the three H19 SNPs and patient age, menopausal status, tumor size, family history, clinical staging, lymph node metastasis, ER status, PR status, HER2 status, triple-negative breast cancer status, P53 status, and BRCA1 and BRCA2 status (P > 0.05).

**Table 4:** Correlations of H19 pomorphisms with clinicopathological parameters in patients with breast cancer.

Characteristic	*r*s11042167 (A→G)				rs2071095 (G→T)				rs2251375 (A→C)			
	AA n (%)	AG/GG n (%)	*P* ^†,‡^	Adjusted OR (95%CI) ^§^	GG n (%)	GT/TT n (%)	*P* ^†,‡^	Adjusted OR (95%CI) ^§^	AA n (%)	AC/CC n (%)	*P* ^†,‡^	Adjusted OR (95%CI) ^§^
Age, yrs												
<50	21 (6.8)	287 (93.2)	0.946	1 (Reference)	133 (43.2)	175 (56.8)	0.073	1 (Reference)	48 (15.6)	260 (84.4)	0.861	1 (Reference)
≥50	19 (7.0)	254 (93.0)	0.784	0.86 (0.313-2.402)	98 (35.9)	175 (64.1)	0.093	1.581 (0.926-2,700)	44 (16.1)	229 (83.9)	0.540	0.802 (0.396-1.624)
Menopausal status												
Premenopausal	22 (7.0)	293 (93.0)	0.918	1 (Reference)	131 (41.6)	184 (58.4)	0.327	1 (Reference)	51 (16.1)	264 (83.8)	0.798	1 (Reference)
Postmenopausal	18 (6.8)	248 (93.2)	0.797	1.143 (0.412-4.169)	100 (37.6)	166 (62.4)	0.508	0.835 (0.489-1.426)	41 (15.4)	225 (84.6)	0.534	1.251 (0.618-2.531)
Tumor size (cm)												
≤ 2.0	19 (7.6)	232 (92.4)	0.761	1 (Reference)	97 (38.6)	154 (61.4)	0.695	1 (Reference)	39 (15.5)	212 (84.5)	0.577	1 (Reference)
2.1-5.0	16 (6.8)	220 (93.2)	0.446	1.485 (0.537-4.103)	93 (39.4)	143 (60.6)	0.385	0.807 (0.497-1.309)	41 (17.4)	195 (82.6)	0.514	1.261 (0.629-2.531)
>5.0	5 (5.3)	89 (94.7)	0.603	1.316 (0.467-3.708)	41 (43.6)	53 (56.4)	0.476	1.133 (0.515-1.363)	12 (12.8)	82 (87.2)	0.293	1.451 (0.725-2.905)
Histology												
IDC	35 (7.0)	464 (93.0)	0.774	1 (Reference)	201 (40.3)	289 (59.7)	0.457	1 (Reference)	77 (15.4)	422 (84.6)	0.324	1 (Reference)
ILC	2 (9.1)	20 (90.9)	0.533	1.471 (0.437-4.946)	6 (27.3)	16 (72.7)	0.961	0.986 (0.569-1.709)	6 (27.3)	16 (72.7)	0.908	1.045 (0.494-2.213)
Others	3 (5.0)	57 (95.0)	0.474	1.980 (0.306-12.823)	24 (40.0)	36 (60.0)	0.231	0.516 (0.175-1.521)	9 (15.0)	51 (85.0)	0.202	2.157 (0.662-7.029)
Clinical stages												
I or II	35 (7.4)	437 (92.6)	0.293	1 (Reference)	191 (40.5)	281 (59.5)	0.469	1 (Reference)	70 (14.8)	402 (85.2)	0.168	1 (Reference)
III or IV	5 (4.6)	104 (95.6)	0.257	1.748 (0.666-4.590)	40 (36.7)	69 (63.3)	0.582	1.130(0.732-1.745)	22 (20.8)	87 (79.8)	0.183	0.695 (0.407-1.187)
Lymph node metastasis status												
Node-negative	28 (8.6)	298 (91.4)	0.067	1 (Reference)	125 (38.3)	201 (61.7)	0.431	1 (Reference)	60 (18.4)	266 (81.6)	0.055	1 (Reference)
Node-positive	12 (4.7)	243 (95.3)	0.074	1.895 (0.941-3.815)	106 (41.6)	149 (58.4)	0.406	0.867 (0.619-1.214)	32 (12.5)	223 (87.5)	0.060	1.564 (0.981-2.493)
ER status												
Negative	17 (6.9)	230 (93.1)	0.936	1 (Reference)	101 (40.9)	146 (59.1)	0.488	1 (Reference)	38 (15.4)	209 (84.6)	0.777	1 (Reference)
Positive	23 (7.1)	303 (92.9)	0.903	0.960 (0.499-1.847)	124 (38.0)	202 (62.0)	0.417	1.152 (0.819-1.620)	53 (16.3)	273 (83.7)	0.731	0.923 (0.585-1.457)
PR status												
Negative	14 (6.0)	219 (94.0)	0.444	1 (Reference)	92 (39.5)	141 (60.5)	0.952	1 (Reference)	33 (14.2)	200 (85.8)	0.344	1 (Reference)
Positive	26 (7.7)	313 (92.3)	0.431	0.762 (0.388-1.498)	133 (39.2)	206 (60.8)	0.798	1.046 (0.741-1.476)	58 (17.1)	281 (82.9)	0.342	0.798 (0.500-1.272)
HER2 status												
Negative	19 (6.3)	282 (93.7)	0.563	1 (Reference)	121 (40.2)	180 (59.8)	0.678	1 (Reference)	51 (16.9)	250 (83.1)	0.470	1 (Reference)
Positive	20 (7.5)	245 (92.5)	0.564	0.825 (0.429-1.586)	102 (38.5)	163 (61.5)	0.779	1.050 (0.747-1.477)	39 (14.7)	226 (85.3)	0.491	1.174 (0.744-1.852)
ER/PR/HER2 status												
TNBC	5 (5.7)	83 (94.3)	0.603	1 (Reference)	34 (38.6)	54 (61.4)	0.895	1 (Reference)	8 (9.1)	80 (90.9)	0.058	1 (Reference)
Non-TNBC	35 (7.2)	450 (92.7)	0.579	1.316 (0.499-3.470)	191 (39.4)	294 (60.6)	0.989	1.003 (0.627-1.604)	83 (17.1)	402 (82.9)	0.058	2.095 (0.974-4.507)
p53 status												
Negative	15 (5.9)	239 (94.1)	0.300	1 (Reference)	97 (38.2)	157 (61.8)	0.522	1 (Reference)	43 (16.9)	211 (83.1)	0.541	1 (Reference)
Positive	25 (8.2)	281 (91.8)	0.314	0.711 (0.366-1.380)	125 (40.8)	181 (59.2)	0.519	0.849 (0.635-1.258)	46 (15.0)	260 (85.0)	0.535	1.154 (0.733-1.818)
BRCA1 status												
Negative	8 (7.2)	103 (92.8)	0.975	1 (Reference)	40 (36.0)	71 (64.0)	0.340	1 (Reference)	18 (16.2)	93 (83.8)	0.945	1 (Reference)
Positive	31 (7.1)	408 (92.9)	0.923	1.041 (0.464-2.335)	180 (41.0)	259 (59.0)	0.316	0.801 (0.519-1.236)	70 (15.9)	369 (84.1)	0.950	1.018 (0.578-1.794)
BRCA2 status												
Negative	10 (4.6)	209 (95.4)	0.058	1 (Reference)	83 (37.9)	136 (62.1)	0.347	1 (Reference)	40 (18.3)	179 (81.7)	0.170	1 (Reference)
Positive	28 (8.8)	289 (91.2)	0.066	0.497 (0.236-1.047)	133 (42.0)	184 (58.0)	0.304	0.830 (0.582-1.183)	44 (13.9)	273 (86.1)	0.167	1.392 (0.871-2.225)

H19, H19 gene; OR, Odds ratio; CI, Confidence interval; IDC, Invasive ductal carcinoma; ILC, Invasive lobular carcinoma; ER, Estrogen receptor; PR, Progesterone receptor; HER2, Human epidermal growth factor receptor 2; TNBC, Triple-Negative Breast Cancer; p53, Tumor suppressor protein 53; BRCA1, Breast carcinoma type 1 susceptibility protein; BRCA2, Breast carcinoma type 2 susceptibility protein.

^†^P values were calculated from 2-sided chi-square tests or Fisher’s Exact Test.

^‡^P values were calculated by unconditional logistic regression adjusted for age, menopausal status.

^§^OR and 95% CI values were calculated by unconditional logistic regression adjusted for age, menopausal status.

### Correlation analysis between the rs11042167, rs2071095, and rs2251375 SNPs and prognosis of breast cancer patients

**Figure 4:**
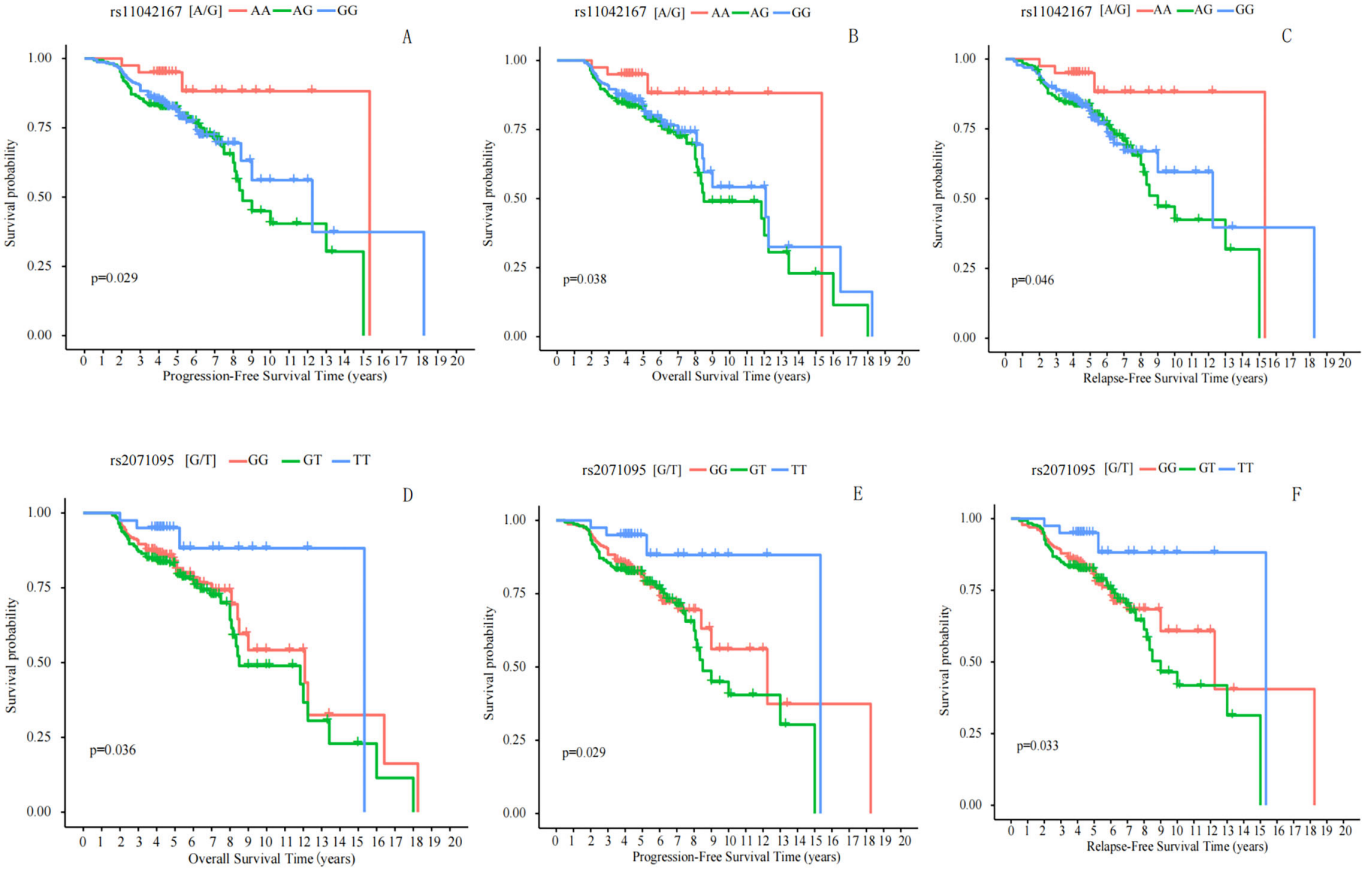
Correlation Analysis of rs11042167, rs2071095, and rs2251375 SNPs with Breast Cancer Prognosis. **(A, E)** Progression-Free Survival: Significant differences in PFS were observed for rs11042167 (A) and rs2071095 (E) SNPs, with patients carrying the AA and TT genotypes, respectively, showing longer survival times. (B, D) Overall Survival: Kaplan - Meier curves for rs11042167 **(B)** and rs2071095 **(D)** reveal that the AA and TT genotypes are associated with improved OS compared to other genotypes. **(C, F)** Recurrence-Free Survival: Significant differences in RFS were found for rs11042167 **(C)** and rs2071095 **(F)**, where AA and TT genotypes were linked to longer recurrence-free periods.

Kaplan−Meier survival analysis was utilized to predict the prognosis of breast cancer patients (n = 581). Progression-Free Survival (PFS) is typically defined as the time from the initial diagnosis of breast cancer to the first occurrence of disease progression or death from any cause. Patients with the AA genotype at rs11042167 SNP had a median PFS of 168 months (95% CI: 146.845–189.283), significantly longer than those with the AG or GG genotypes, who had a median PFS of 122 months (log-rank P = 0.029). At rs2071095, patients with the TT genotype showed a median PFS of 138 months (95% CI: 120.123–155.877), significantly longer than those with the GT or GG genotypes, who had a median PFS of 113 months (log-rank P = 0.036). However, at rs2251375, there was no significant difference in median PFS between patients with the AA genotype (116 months) and those with the AC or CC genotypes (129 months) (log-rank P = 0.795). Please refer to **Figures 4A**, **E**.

Overall Survival (OS) is generally defined as the time from randomization in a study until death from any cause. We analyzed the correlation between the rs11042167, rs2071095, and rs2251375 SNPs and OS in breast cancer patients. We found that in patients with the AA genotype at rs11042167, the median time to death was 189 months (95% CI = 172.833–205.168), while in patients with the AG or GG genotype, the time to death was 136 months, and there was a statistically significant difference between the two groups (log-rank P = 0.038). Similarly, we analyzed rs2071095 and found that there was a statistically significant difference in median time to death between patients with the TT genotype 132 months(95% CI = 114.357–149.643) and those with the GT or GG genotype 108 months (log-rank; P = 0.029).We also analyzed rs2251375 and found that there was no statistically significant difference in median time to death between patients with the AA genotype (132 months) and those with the AC or CC genotype (129 months) (log-rank P = 0.780). Please refer to **Figures 4B**, **D**.

Recurrence-Free Survival (RFS) is typically defined as the time from treatment initiation or randomization until disease recurrence or death from any cause. We analyzed the correlation between three SNPs, rs11042167, rs2071095, and rs2251375, and RFS in breast cancer patients. We found that in patients with the AA genotype at rs11042167, the median time to recurrence was 176 months (95% CI = 160.245–191.755), whereas in those with the AG or GG genotypes, the median time to recurrence was 130 months, showing a statistically significant difference (log-rank P = 0.046). Similarly, we analyzed rs2071095 and found that in patients with the TT genotype, the median time to recurrence was 125 months (95% CI = 109.624–140.376), compared to 98 months in those with the GT or GG genotypes, which also demonstrated a statistically significant difference (log-rank P = 0.033). We also analyzed rs2251375 and found that in patients with the AA genotype, the median time to recurrence was 128 months, compared to 124 months in those with the AC or CC genotypes, with no statistically significant difference between the groups (log-rank P = 0.879). For the results that exhibited statistical significance, please refer to **Figures 4C**, **F**.

We further investigated the relationship between the rs11042167, rs2071095, and rs2251375 SNPs and the survival outcomes, specifically Progression-Free Survival (PFS), Overall Survival (OS), and Recurrence-Free Survival (RFS), across various pathological parameters in breast cancer patients. Our stratified analysis, which accounted for factors such as age, menopausal status, tumor size, family history, clinical stage, lymph node metastasis, estrogen receptor (ER) status, progesterone receptor (PR) status, HER2 status, triple-negative breast cancer prevalence, P53 status, and BRCA1/2 status, revealed no significant correlation between these SNPs and PFS in breast cancer patients (P > 0.05).

### The correlation analysis between SNPs rs11042167, rs2071095, and rs2251375 and the expression of LincRNA H19 and miR-675 in breast cancer tissues

**Figure 5:**
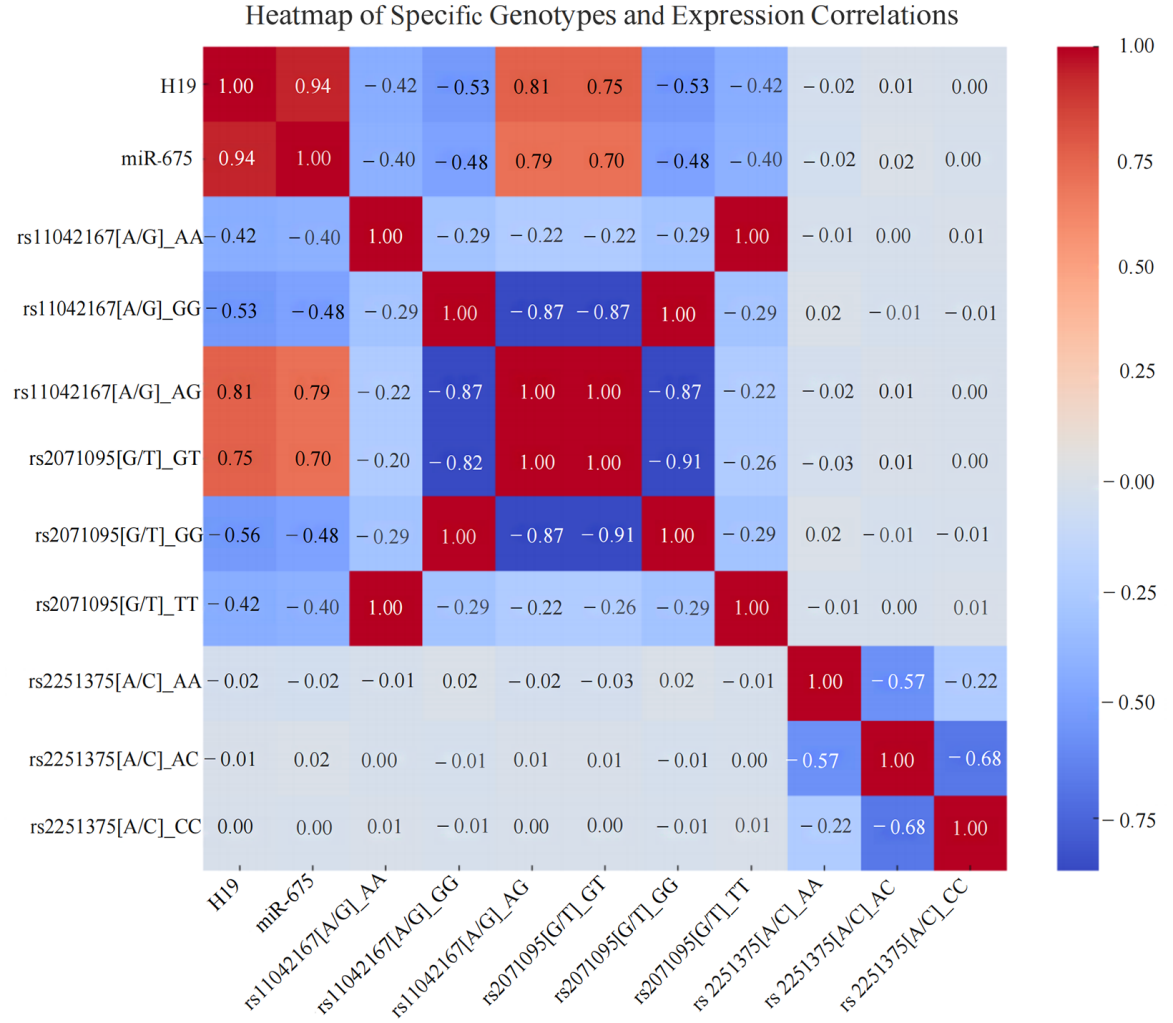
Heatmap of the Correlation Between Different Breast Cancer Genotypes and the Expression of LincRNA H19, miR-675 in Breast Cancer Tissues.

Through Spearman correlation heatmap analysis, it was found that the AG genotype of rs11042167 [A/G] is positively correlated with the expression of LincRNA H19 and miR-675 (P < 0.001). For rs2071095 [G/T], the GT genotype shows a positive correlation with the expression of LincRNA H19 and miR-675 (P < 0.001), while the other genotypes are negatively correlated. For rs2251375 [A/C], Spearman correlation heatmap analysis reveals no significant correlation with the expression levels of LincRNA H19 and miR-675. Additionally, the genotypes rs11042167 [A/G]_AA and rs2071095 [G/T]_TT exhibit a complete positive correlation (P < 0.001), as do the genotypes rs2071095 [G/T]_GT and rs11042167 [A/G]_AG (P < 0.001). See **Figure 5**.

### Expression profiles and correlation analysis of LincRNA H19, miR-675, MRP3, HOXA1, and MMP16 in breast cancer tissues

*In situ* hybridization results revealed significant expression differences of LincRNA H19 in breast cancer tissues and adjacent non-tumor tissues. Out of 581 breast cancer samples, 563 showed high expression of LincRNA H19 and 18 exhibited low expression. In contrast, in the adjacent non-tumor tissues (n = 581), 35 samples displayed high expression and 546 showed low expression, demonstrating statistical significance (P < 0.01). Similarly, miR-675 was highly expressed in 552 of the breast cancer tissues and lowly expressed in 29, while in the adjacent non-tumor tissues, high expression was noted in only 16 samples and low expression in 565 samples, also with significant differences (P < 0.01).

Immunohistochemistry results were consistent with these findings. High expression of MRP3 was observed in 563 out of 581 breast cancer tissues, with low expression in 18, whereas among the adjacent tissues, only 35 showed high expression compared to 546 with low expression, indicating significant differences (P < 0.01). For HOXA1, high expression was noted in 552 cancerous samples and low expression in 29, with the adjacent tissues showing high expression in only 16 samples and low in 565 samples, which was statistically significant (P < 0.01). A similar pattern was observed for MMP16, with high expression in 552 breast cancer tissues and low expression in 29, while in the adjacent tissues, high expression was noted in 16 samples and low in 565 samples, indicating significant differences (P < 0.01).

**Figure 6:**
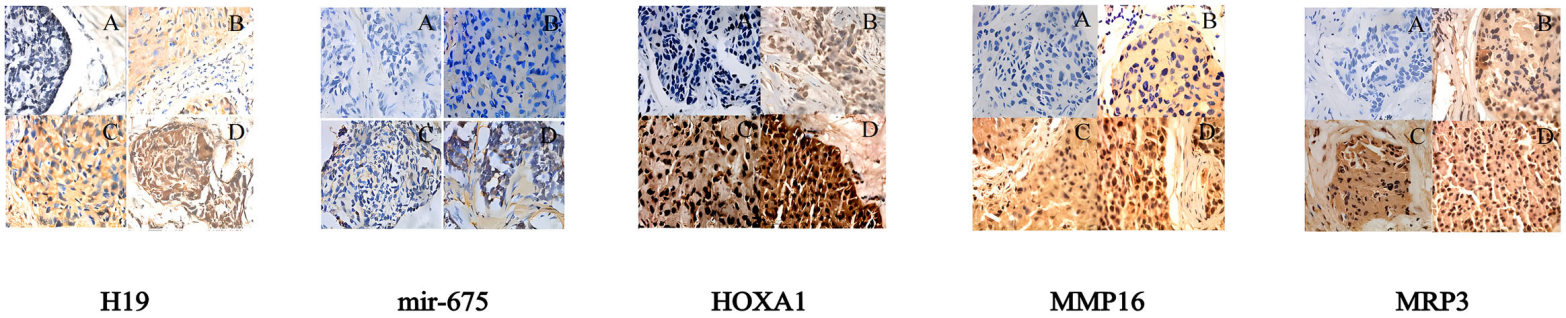
Pathological test results for LincRNA H19, miR-675, MRP3, HOXA1, and MMP16 (400x magnification): **(A)** Negative expression in breast cancer tissues; **(B)** Low expression in breast cancer tissues; **(C)** Moderate expression in breast cancer tissues; **(D)** High expression in breast cancer tissues.

**Figure 7:**
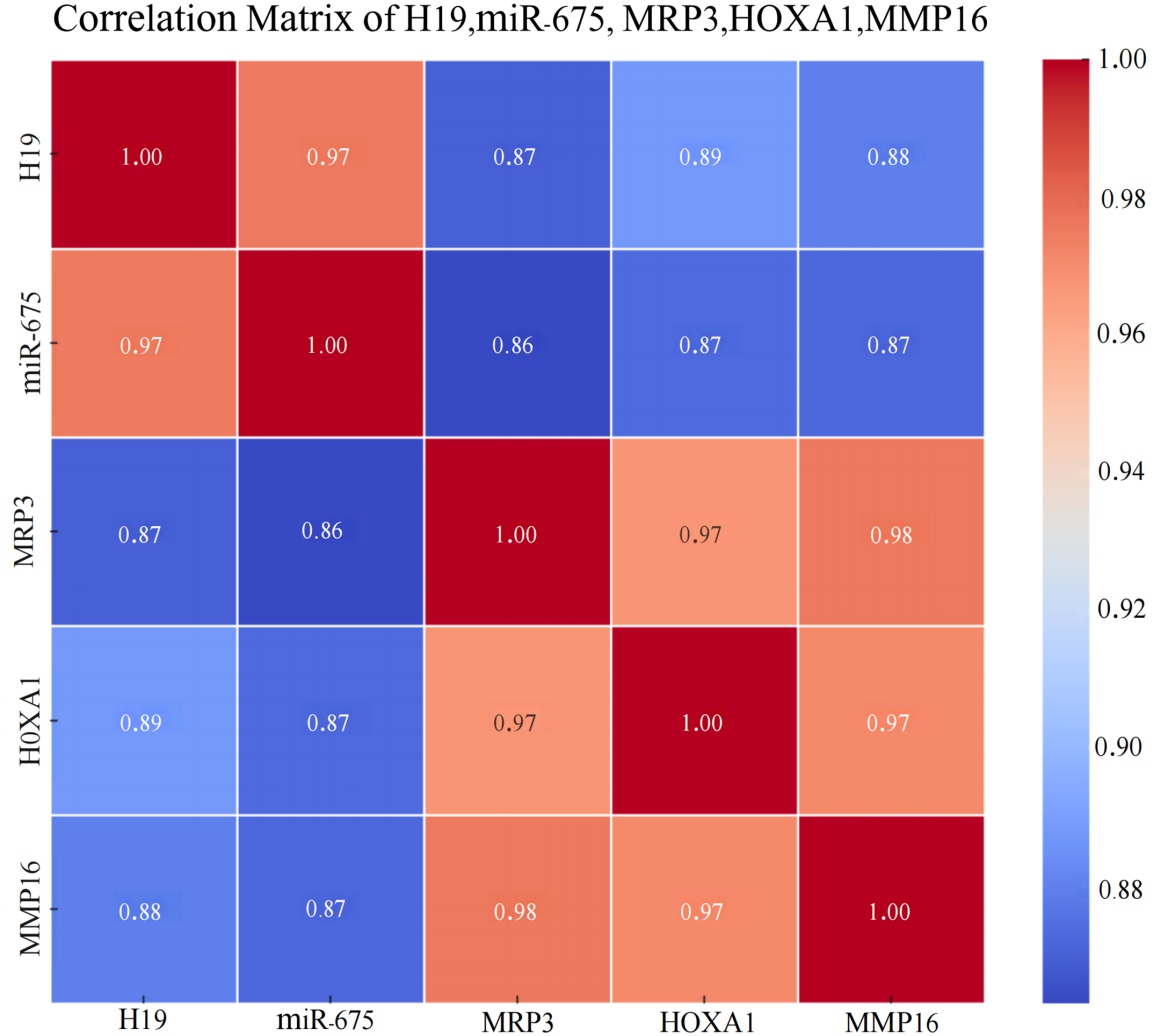
Heatmap of the Correlation Among LincRNA H19, miR-675, MRP3, HOXA1, and MMP16 in Breast Cancer Tissues.

Furthermore, Spearman correlation heatmap analysis revealed a strong positive correlation among the expressions of LincRNA H19, miR-675, MRP3, HOXA1, and MMP16 in breast cancer tissues. The detailed results of the *in situ* hybridization and immunohistochemistry are depicted in **Figure 6**, with the correlation heatmap shown in **Figure 7**. (The numerical values on the image that are closer to 1 indicate a stronger correlation, See **Table 5** for more details).

**Table 5:** Positive expression rates of LincRNA H19, miR-675, MRP3, HOXA1, and MMP16 across various tissues.

Biomarkers	Breast cancer tissue n (%)	Adjacent non-cancerous tissue n (%)	χ2	P^†^
LincRNA H19			487.9	<0.0001
+	563 (94.1)	35 (5.9)		
−	18 (3.1)	546 (96.9)		
miR-675			672.2	<0.0001
+	552 (97.1)	16 (2.9)		
−	29 (4.8)	565 (95.2)		
MRP3			145.6	<0.0001
+	513 (94.8)	28 (5.2)		
−	68 (10.9)	553 (89.1)		
HOXA1			908.8	<0.0001
+	566 (96.2)	22 (3.8)		
−	15 (2.6)	559 (97.4)		
MMP16			179.7	<0.0001
+	538 (93.5)	37 (6.5)		
−	43 (7.3)	544 (92.7)		

^†^P values were calculated from 2-sided chi-square tests or Fisher’s Exact Test.

### The association between the expression levels of LincRNA H19, miR-675, MRP3, HOXA1, and MMP16 and clinical pathological parameters in breast cancer

The expression of LincRNA H19 is associated with age, clinical stages, histology, ER status, HER2 status, and p53 status (P < 0.05), but not with other clinical pathological parameters. miR-675 expression correlates with age, menopausal status, tumor size, histology, clinical stages, ER status, HER2 status, non-TNBC, BRCA1 status, and BRCA2 status (P<0.05), but not with other clinical pathological parameters. MRP3 expression is associated with menopausal status, tumor size, histology, clinical stages, lymph node metastasis status, PR status, HER2 status, p53 status, and BRCA2 status (P < 0.05), but not with other clinical pathological parameters. HOXA1 expression correlates with histology, clinical stages, BRCA1 status, and BRCA2 status (P < 0.05), but not with other clinical pathological parameters. MMP16 expression is associated with age, tumor size, histology, clinical stages, lymph node metastasis status, PR status, HER2 status, non-TNBC, and BRCA1 status (P < 0.05), but not with other clinical pathological parameters. These associations are detailed in **Table 6**.

**Table 6:** Correlations of LincRNA H19, miR-675, MRP3, HOXA1, and MMP16 with clinicopathological parameters in patients with breast cancer.

Characteristic	*LincRNA H19*			miR-675			MRP3			HOXA1			MMP16		
	+	-	*P* ^†^	+	-	*P* ^†^	+	-	*P* ^†^	+	-	*P* ^†^	+	-	*P* ^†^
**Age, yrs**			**0.0326**			**0.0018**			>0.9999			0.4329			**0.0384**
<50	303 (98.3)	5 (1.7)		301 (97.7)	7 (2.3)		272 (88.3)	36 (11.7)		302 (98.0)	6 (2.0)		292 (94.8)	16 (5.2)	
≥50	260 (95.3)	13 (4.7)		251 (91.9)	22 (8.1)		241 (88.2)	32 (11.8)		264 (96.7)	9 (3.3)		246 (90.1)	27 (9.9)	
**Menopausal status**			0.8117			**0.0207**			**<0.0001**			0.0632			0.6358
Premenopausal	306 (97.1)	9 (2.9)		299 (97.0)	9 (3.0)		263 (83.4)	52 (16.6)		303 (96.1)	12 (3.9)		290 (92.0)	25 (8.0)	
Postmenopausal	257 (96.6)	9 (3.4)		253 (92.6)	20 (7.4)		289 (94.7)	16 (5.3)		263 (98.8)	3 (1.2)		248 (93.2)	18 (6.8)	
**Tumor size (cm)**			0.6624			**<0.0001**			**<0.0001**			>0.9999			**<0.0001**
≤ 2.0	245 (97.6)	6 (2.4)		249 (99.2)	2 (0.8)		244 (97.2)	7 (2.8)		247 (98.4)	4 (1.6)		248 (98.8)	3 (1.2)	
2.1-5.0	227 (96.1)	9 (3.9)		230 (97.4)	6 (2.6)		214 (90.6)	22 (9.4)		228 (96.6)	8 (3.4)		221 (93.6)	15 (6.4)	
>5.0	91(96.8)	3(3.2)		73 (77.6)	21 (22.4)		55 (58.5)	39 (41.5)		91 (96.8)	3 (3.2)		69 (73.4)	25 (26.6)	
**Histology**			**<0.0001**			**<0.0001**			**<0.0001**			**<0.0001**			**<0.0001**
IDC	491 (98.3)	8 (1.7)		490 (98.1)	9 (1.9)		481 (96.3)	18 (3.7)		481 (98.3)	8 (1.7)		471 (94.3)	28 (5.7)	
ILC	15 (68.1)	7 (31.9)		4 (18.1)	18 (81.9)		7 (31.8)	15 (68.2)		16 (72.7)	6 (28.3)		11 (50.0)	11 (50.0)	
Others	57 (95.0)	3 (5.0)		58 (96.6)	2 (3.4)		25 (41.6)	35 (58.4)		59 (98.3)	1 (1.7)		56 (93.3)	4 (6.7)	
**Clinical stages**			**0.0128**			**0.0008**			**<0.0001**			**<0.0001**			**<0.0001**
I or II	481 (97.8)	11 (2.2)		456 (96.6)	16 (3.4)		445 (94.2)	27 (5.8)		469 (99.3)	3 (0.7)		454 (96.1)	18 (3.9)	
III or IV	82 (92.1)	7 (7.9)		96 (88.0)	13 (12.0)		68 (62.3)	41 (37.7)		97 (88.9)	12 (11.1)		84 (77.0)	25 (33.0)	
**Lymph node metastasis status**			>0.9999			0.1809			**<0.0001**			0.5993			**<0.0001**
Node-negative	316 (96.9)	10 (3.1)		306 (93.8)	20 (6.2)		308 (94.4)	18 (5.6)		321 (96.9)	10 (3.1)		290 (88.9)	36 (11.1)	
Node-positive	247 (96.8)	8 (3.2)		246 (96.4)	9 (3.6)		205 (80.3)	50 (19.7)		245 (98.0)	5 (2.0)		248 (97.2)	7 (2.8)	
**ER status**			**0.0146**			**0.0008**			0.5156			0.1965			>0.9999
Negative	234 (94.7)	13 (5.3)		243 (98.3)	4 (1.7)		215 (87.0)	32 (13.0)		238 (96.3)	9 (3.7)		229 (92.7)	18 (7.3)	
Positive	321 (98.4)	5 (1.6)		301 (92.3)	25 (7.7)		290 (88.9)	36 (11.1)		320 (98.1)	6 (1.9)		301 (92.3)	25 (7.7)	
**PR status**			0.3326			0.1751			**0.0025**			0.6067			**<0.0001**
Negative	228 (97.8)	5 (2.2)		225 (96.5)	8 (3.5)		211 (90.5)	22 (9.5)		228 (97.8)	5 (2.2)		201 (86.2)	32 (13.8)	
Positive	326 (96.1)	13(3.9)		318 (93.8)	21 (6.2)		193 (80.7)	46 (19.3)		329 (97.0)	10 (3.0)		315 (96.6)	11 (3.4)	
**HER2 status**			**0.0028**			**0.0004**			**0.0197**			>0.9999			**0.0379**
Negative	298 (99.0)	3 (1.0)		295 (98.0)	6 (2.0)		274 (91.0)	27 (9.0)		293 (97.3)	8 (2.7)		285 (94.6)	16 (5.4)	
Positive	250 (94.3)	15(5.7)		242 (91.3)	23 (8.7)		224 (84.5)	41 (15.5)		258 (97.3)	7 (2.7)		238 (89.8)	27 (10.2)	
**ER/PR/HER2 status**			0.5010			**<0.0001**			0.2489			>0.9999			**<0.0001**
TNBC	84 (95.4)	4 (4.6)		73 (82.9)	15 (17.1)		76 (86.3)	12 (13.7)		86 (97.7)	2 (2.3)		66 (75.0)	22 (25.0)	
Non-TNBC	471 (97.1)	14 (2.9)		471 (97.1)	14 (2.9)		439 (90.5)	46 (9.5)		472 (97.3)	13 (2.7)		464 (95.6)	21 (4.4)	
**p53 status**			**0.0001**			0.1797			**0.0131**			0.7953			>0.9999
Negative	238 (93.7)	16 (6.3)		237 (93.3)	17 (6.7)		233 (91.7)	21 (8.3)		248 (97.6)	6 (2.4)		235 (92.5)	19 (7.5)	
Positive	304 (99.3)	2 (0.7)		294 (96.0)	12 (4.0)		259 (84.6)	47 (15.4)		297 (97.0)	9 (3.0)		282 (92.1)	24 (7.9)	
**BRCA1 status**			>0.9999			**<0.0001**			0.1362			**0.0040**			**<0.0001**
Negative	108 (97.2)	3 (2.8)		91 (81.9)	20 (19.1)		98 (88.2)	13 (11.8)		103 (92.7)	8 (7.3)		79 (71.1)	32 (28.9)	
Positive	424 (96.5)	15 (3.5)		430 (97.9)	9 (2.1)		251 (82.0)	55 (18.0)		432 (98.4)	7 (1.6)		306 (96.5)	11 (3.5)	
**BRCA2 status**			0.4693			**0.0311**			**0.0244**			**0.0322**			0.5180
Negative	210 (95.8)	9 (4.2)		213 (97.2)	6 (2.8)		200 (91.3)	19 (8.7)		217 (99.0)	2 (1.0)		199 (90.8)	20 (9.2)	
Positive	308 (97.1)	9 (2.9)		294 (92.7)	23 (7.3)		268 (84.5)	49 (15.5)		304 (95.8)	13 (4.2)		294 (92.7)	23 (7.3)	

H19, H19 gene; OR, Odds ratio; CI, Confidence interval; IDC, Invasive ductal carcinoma; ILC, Invasive lobular carcinoma; ER, Estrogen receptor; PR, Progesterone receptor; HER2, Human epidermal growth factor receptor 2; TNBC,Triple-Negative Breast Cancer; p53, Tumor suppressor protein 53; BRCA1, Breast carcinoma type 1 susceptibility protein; BRCA2, Breast carcinoma type 2 susceptibility protein.

^†^Bold values indicate statistically significant results (P < 0.05) from 2-sided chi-square tests or Fisher's Exact Test, highlighting important findings in the correlation of LincRNA H19, miR-675, MRP3, HOXA1, and MMP16 with clinicopathological parameters in breast cancer patients.

### The association between the expression levels of LincRNA H19, miR-675, MRP3, HOXA1, and MMP16 and the prognosis in breast cancer patients

**Figure 8:**
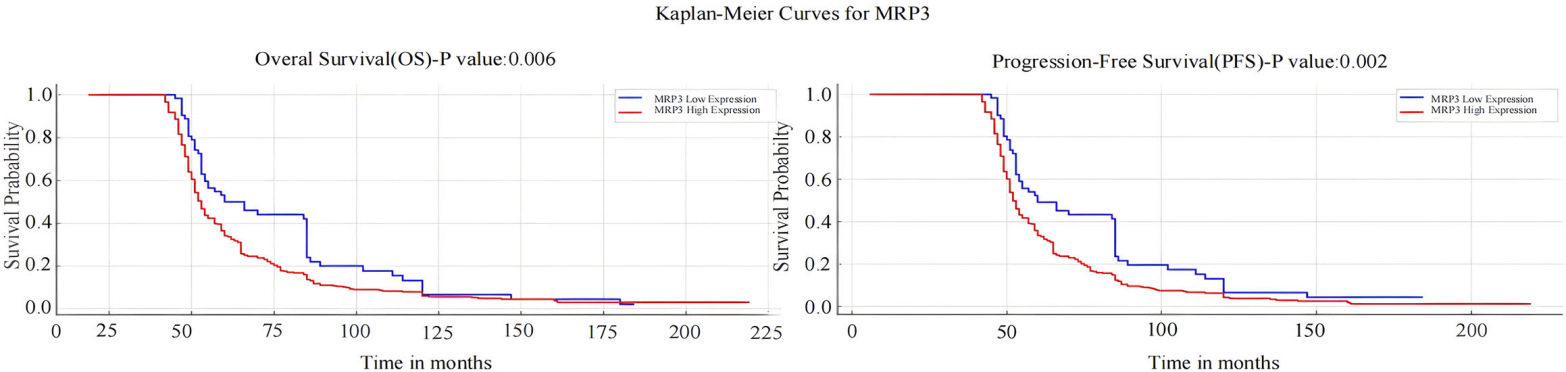
Kaplan-Meier Survival Analysis of MRP3 Expression with Overall Survival (OS) and Progression-Free Survival (PFS) in Breast Cancer Patients.

Kaplan-Meier analysis revealed that breast cancer patients with high MRP3 expression have shorter overall survival (OS) and progression-free survival (PFS) compared to those with low MRP3 expression, a difference that is statistically significant (P < 0.05). See **Figure 8**. Surprisingly, the expression levels of other markers were not associated with the prognosis of breast cancer patients (P > 0.05).

## Discussion

In recent years, the “common disease, common variant” hypothesis has frequently been mentioned in research on disease diagnosis and treatment (28–31). This hypothesis suggests that susceptibility to certain diseases is caused by variations at genetic loci, particularly variations in gene regulatory or coding regions. Comparisons of SNP sites between a malignant tumor population and a normal control population allow correlations between SNP sites and the risk of tumor incidence to be determined and applied to the study of genetic susceptibility to tumors. With the successful completion of the Human Genome Project, the focus of life science research is rapidly shifting toward understanding the function of each gene, the interactions between multiple genes and their products (32–36), and the interactions between the genome and the environment (37–39). The study of SNPs within the sequence of the human genome has become the focus of a new round of genome science research (40–44).

Polymorphisms in the H19 gene have been suggested to play very important roles in the occurrence and development of tumors (45). Ayesh et al. found that lncRNA H19 upregulates many genes that are closely related to the invasion, migration, and angiogenesis of tumor cells (46). Tanos et al. found that overexpression of H19 is important for the growth of esophageal and colorectal cancer cells (47). Berteaux et al. also found that H19 overexpression in breast cancer tissue promotes cell proliferation (48). A few studies have shown that H19 may act as a tumor suppressor gene and participate in mediating human growth and development (49–51). However, the appearance of genetic variations may cause abnormal expression of H19, which leads to the disruption of gene regulation mechanisms and increases the risk of tumor occurrence (52–54).

In this study, the rs11042167, rs2071095, and rs2251375 SNPs were found to be associated with breast cancer risk in both the case and control groups. At the rs11042167 SNP, individuals with the AG heterozygous genotype had an increased risk of disease compared to those with the AA genotype. At the rs2071095 SNP, individuals with the GT heterozygous genotype had an increased risk of disease when compared to those with the GG genotype. At the rs2251375 SNP, individuals with the AC heterozygous genotype had an increased risk of disease when compared to those with the AA genotype. This suggested that heterozygous genotypes at the rs11042167, rs2071095, and rs2251375 SNPs may be risk factors for breast cancer in the Chinese population. However, these SNPs did not show any correlation to breast cancer with different pathological parameters. In a survival analysis, it was found that patients carrying the AA genotype at the rs11042167 SNP had an increased PFS and OS, which indicated a better prognosis. In contrast, rs2071095 and rs2251375 did not show any correlation with survival in the overall breast cancer patient population. The rs2071095 and rs2251375 SNPs did not show statistically significant differences in PFS and OS in breast cancer patients stratified by pathological status.

In this study, the expression levels of LincRNA H19, miR-675, MRP3, HOXA1, and MMP16 were consistently higher in 581 breast cancer tissues compared to adjacent non-tumor tissues. This significant upregulation suggests a pivotal role for LincRNA H19 as an oncogene in the progression and prognosis of breast cancer. Additionally, it indicates the potential of LincRNA H19 and miR-675 as diagnostic and prognostic markers and therapeutic targets. The study also found that high expression levels of LincRNA H19, miR-675, MRP3, HOXA1, and MMP16 were more common in patients with early-stage breast cancer (TNM stages I or II) and those diagnosed with invasive ductal carcinoma (IDC), suggesting their utility as early diagnostic markers to facilitate the early detection and treatment of IDC.

Furthermore, Spearman correlation heatmap analysis showed a positive correlation between the AG genotype of rs11042167 [A/G] and the expression of LincRNA H19 and miR-675 (P < 0.001), and between the GT genotype of rs2071095 [G/T] and the expression of LincRNA H19 and miR-675 (P < 0.001), with other genotypes showing negative correlations. However, no significant correlation was observed for rs2251375 [A/C], likely due to the complexity of gene regulation, genetic background variability, environmental factors, and the biological heterogeneity of cancer.

Moreover, the study explored the relationship between the expression of these markers and survival in breast cancer tissues, assessing their prognostic value. Results indicated that higher MRP3 expression correlates with shorter overall survival (OS) and disease-free survival (DFS), suggesting poorer prognosis for patients with high expression levels, and underscoring their relevance in prognosis assessment.

Genetic correlation analysis identified complete positive correlations (correlation coefficient of 1) among several genotypes, indicating complete linkage disequilibrium. Specifically, the AA genotype of rs11042167 [A/G] correlated perfectly with the TT genotype of rs2071095 [G/T], and similarly, the GG genotypes of both rs11042167 and rs2071095 showed a correlation coefficient of 1. Additionally, the GT genotype of rs2071095 and the AG genotype of rs11042167 were also perfectly correlated (P < 0.001). This strong linkage suggests that these loci may co-regulate H19 expression, potentially influencing transcription factor binding. Given the association of H19 with the development of various cancers, these variants are potentially crucial for studying disease mechanisms and clinical diagnostics, warranting further investigation into their precise functional roles and their impact on disease progression.

In addition to the findings on SNPs and LincRNA H19, this study also investigated the expression of MRP3, HOXA1, and MMP16 in breast cancer tissues. MRP3 expression did not vary significantly with age (P>0.9999) but showed significant differences with menopausal status, tumor size, histology, clinical stage, lymph node metastasis, PR status, and BRCA2 status. Specifically, MRP3 expression was lower in premenopausal compared to postmenopausal patients (P<0.0001), higher in tumors ≤2.0 cm (P<0.0001), and higher in IDC compared to ILC (P<0.0001). MRP3 was also more frequently expressed in early-stage (I/II) than late-stage (III/IV) cancers (P<0.0001) and in lymph node-negative patients (P<0.0001). PR-positive patients showed higher MRP3 expression (P=0.0025), and BRCA2-positive patients had higher MRP3 levels (P=0.0244).

For HOXA1, no significant age-related differences were observed (P=0.4329), but significant differences were found in tumor size, histology, clinical stage, ER status, and BRCA1 status. HOXA1 was more frequently expressed in smaller tumors (≤2.0 cm), particularly in IDC (P<0.0001), and in early-stage cancers (P<0.0001). Its expression was higher in BRCA1-positive patients (P=0.0040), indicating its potential role in breast cancer progression.

MMP16 expression varied with age, tumor size, histology, clinical stage, lymph node metastasis, and PR status. It was higher in younger patients (P=0.0384), smaller tumors (≤2.0 cm, P<0.0001), IDC (P<0.0001), early-stage cancers (P<0.0001), lymph node-positive patients (P<0.0001), and PR-positive patients (P<0.0001), suggesting its involvement in tumor invasiveness and metastasis. These findings reveal the potential roles of MRP3, HOXA1, and MMP16 in breast cancer, highlighting their significance as prognostic markers and potential therapeutic targets.

## Conclusions

In summary, the heterozygous AG genotype at rs11042167, the GT genotype at rs2071095, and the AC genotype at rs2251375 were associated with an increased susceptibility to breast cancer. Additionally, the AA genotype at rs11042167 and the TT genotype at rs2071095 were correlated with a favorable prognosis for breast cancer patients. The expression levels of LincRNA H19, miR-675, MRP3, HOXA1, and MMP16 in breast cancer tissues also suggest a strong link with the early onset and types of breast cancer, with MRP3 showing high prognostic value. Furthermore, the AG genotype at rs11042167 and the GT genotype at rs2071095 were positively correlated with the expression of LincRNA H19 and miR-675 (P < 0.001), supporting the potential of the H19/LincRNA H19/miR-675/MRP3-HOXA1-MMP16 axis as a new direction for targeted therapy in breast cancer. LincRNA H19 and miR-675 are also promising as new diagnostic markers for breast cancer.

## Data Availability

The datasets presented in this study can be found in online repositories. The names of the repository/repositories and accession number(s) can be found in the article/**Supplementary Material**.
