# Supplementary material for: Influence of H19 polymorphisms on breast cancer: risk assessment and prognostic implications via LincRNA H19/miR-675 and downstream pathways
Source: Front Oncol. 2024 Aug 29;14:1436874. doi: 10.3389/fonc.2024.1436874 (PMC11390531; doi:10.3389/fonc.2024.1436874)
Supplement: Supplementary file 2 [file Table1.docx]

The TaqMan probes used for allelic discrimination were

rs11042167, 5′-TTGGGGGCTGTCCTTAGACGGAGTC[A/G]GAGCTGTGCTCTGGGATAGATGTGG-3′, where the A allele was detected by the HEX labeled probe and the G allele by the FAM labeled probe;

rs2071095, 5′-CCTGCGGCTTGTGGACTCGGTACGG[G/T]GTGGGGATCCTGATGGGG TTAGGAT-3′, where the G allele was detected by the HEX labeled probe and the T allele by the FAM labeled probe;

rs2251375, 5′-GTTTAGTGATTTACTGCTTACAAAG[C/G]ACTTACACACCTTATACACTCCCTG-3′, where the C allele was detected by the HEX-labeled probe and the G allele by the FAM-labeled probe.
